# An HIV Vaccine for South-East Asia—Opportunities and Challenges

**DOI:** 10.3390/vaccines1030348

**Published:** 2013-08-14

**Authors:** Punnee Pitisuttithum, Supachai Rerks-Ngarm, Robert J. O’Connell, Jerome H. Kim, Jean-Louis Excler

**Affiliations:** 1 Vaccine Trial Center, Faculty of Tropical Medicine, Mahidol University, 420/6 Rajvithi Road, Bangkok 10400, Thailand; E-Mail: tmppt@mahidol.ac.th; 2 Department of Disease Control, Ministry of Public Health, Nonthaburi 11000, Thailand; E-Mails: supachai@health.moph.go.th or supachair@biophics.org; 3 Department of Retrovirology, US Army Medical Component, Armed Forces Institute of Medical Sciences (AFRIMS), 315/6 Rajvithi Road, Bangkok 10400, Thailand; E-Mail: robert.oconnell@afrims.org; 4 U.S. Military HIV Research Program (MHRP), Walter Reed Army Institute of Research, 6720-A Rockledge Drive, Suite 400, Bethesda, MD 20817, USA; E-mail: Jkim@hivresearch.org

**Keywords:** HIV vaccines, RV144, correlates of risk, efficacy trials, HIV-1 subtypes, Men who have sex with men, South-East Asia, Thailand, Myanmar, China

## Abstract

Recent advances in HIV vaccine development along with a better understanding of the immune correlates of risk have emerged from the RV144 efficacy trial conducted in Thailand. Epidemiological data suggest that CRF01_AE is still predominant in South-East Asia and is spreading in China with a growing number of circulating recombinant forms due to increasing human contact, particularly in large urban centers, tourist locations and in sites of common infrastructure. A vaccine countering CRF01_AE is a priority for the region. An Asia HIV vaccine against expanding B/E or BCE recombinant forms should be actively pursued. A major challenge that remains is the conduct of efficacy trials in heterosexual populations in this region. Men who have sex with men represent the main target population for future efficacy trials in Asia. Coupling HIV vaccines with other prevention modalities in efficacy trials might also be envisaged. These new avenues will only be made possible through the conduct of large-scale efficacy trials, interdisciplinary teams, international collaborations, and strong political and community commitments.

## 1. Introduction

South-East Asia (SEA) is home to more than 593 million people. Economic and tourism exchanges within SEA, and between SEA and East Asia, especially China, are thriving and expected to intensify over the next decade [1]. The further development of roads, communications and other major infrastructures promises to intensify the interchange between China and SEA, and the emergence of Myanmar after decades’ long isolation will generate new economic activity and the potential for a geographically continuous market that includes India, China and SEA Asia—constituting nearly 3 billion people.

Aggressive HIV prevention measures and expanded access to care and treatment for HIV-infected individuals [2,3] have yielded a 20% decline in new HIV infections from 450,000 in 2001 to 360,000 in 2009, with approximately 4.9 million individuals living with HIV in Asia and the Pacific. Southern Chinese provinces, Cambodia, Indonesia, Malaysia, Myanmar, Papua New Guinea, Thailand and Viet Nam remain the most affected in the region. However, the epidemic still outpaces the response with almost two new HIV infections for every person who starts treatment. Most countries are far from achieving universal access targets for HIV prevention, treatment, care and support [4]. A preventive HIV vaccine as part of a comprehensive prevention package [5,6] remains therefore among the best hopes for controlling the HIV/AIDS epidemic in the region [7,8]. Thailand’s outstanding achievements in HIV prevention [9,10], care and treatment, and HIV vaccine development [11] have paved the way to a regional approach for a preventive HIV vaccine.

## 2. Opportunities

### 2.1. HIV Vaccine Clinical Development

Considerable HIV vaccine clinical development efforts have been deployed in the region since the mid 1990s [12]. Key studies were conducted in Thailand, which advanced the field considerably. The first Phase III trial in a middle-income country (Vax003) tested a bivalent recombinant gp120 B/E (MN and A244 CRF01_AE) adjuvant in alum (AIDSVAX® B/E) in injecting drug users (IDU) in Bangkok, Thailand [13]. No difference in HIV incidence was detected between the vaccine and placebo arms. HIV-1 CRF01_AE accounted for 77% of infections. No statistically significant effects of the vaccine on plasma HIV-1 load, CD4 cell count, onset of acquired immunodeficiency syndrome-defining conditions, or initiation of antiretroviral therapy secondary end points were observed. 

A community-based Phase III trial (RV144) provided the first evidence that an HIV-1 vaccine might prevent HIV infection [14,15]. The prime-boost vaccine regimen consisted of a recombinant canarypox vector, ALVAC-HIV (vCP1521) prime, expressing *gag*, *protease* subtype B (LAI) and *env* gp120 TH023 CRF01_AE genes with a gp41 subtype B (LAI) transmembrane anchor, administered at 0, 1, 3, and 6 months and a AIDSVAX® B/E boost given at months 3 and 6. The vaccine regimen was safe and generally well tolerated [16]. The modified intent-to-treat analysis showed 31.2% efficacy after 42 months of follow-up. There was no effect on early post-infection HIV-1 RNA viral load or CD4+ T-cell count. In a *post-hoc* analysis, vaccine efficacy appeared to be higher (60%) at 12 months post vaccination, suggesting an early, but nondurable, vaccine effect. RV144 was not designed and powered to assess the interaction of vaccine efficacy and risk behavior. HIV risk behavior was assessed with a self-administered questionnaire at the time of initial vaccination in the trial and every six months thereafter for three years. In a post-hoc analysis, participants classified as high or increasing risk at least once during follow-up were compared with those who maintained low-risk or medium-risk behavior as a time-varying covariate; the interaction of risk status and acquisition efficacy showed a greater benefit in low-risk individuals. The authors pointed out that future HIV vaccine trials should recognize potential interactions between challenge intensity and risk heterogeneity in both population and treatment effects [17]. One can speculate that the RV144 regimen was able to protect against infection because the number of sexual contacts were limited in time and could be countered by a marginally efficacious vaccine, which might or might not hold true with communities at higher risk of sexual transmission and increased number of exposures such as MSM or female sex workers. This remains to be demonstrated in future trials using improved immunogens and additional boosts. An analysis of the effect of vaccination on disease progression after infection showed weak evidence of lower viral load and higher CD4+ count in the vaccine group. Vaccination did not affect the clinical course of HIV disease after infection. Interestingly, lower mucosal viral load was observed among vaccine recipients, primarily in semen, suggesting a vaccine-induced effect caused by mucosal immune responses differing from those measured in the peripheral blood. Moreover, a lower RNA viral load in mucosal secretions of vaccine recipients could translate into lower transmission, a potential public health benefit [18].

The efficacy observed in the RV144 trial provided the first opportunity to study immune correlations associated with vaccine efficacy against HIV. A case-control study showed that IgG antibodies to the scaffolded V1/V2 region of HIV-1 gp120 correlated with decreased risk of infection while IgA antibodies to the envelope correlated with decreased vaccine efficacy in the vaccine group [19,20,21], but no responses were associated with enhancement of HIV-1 infection risk. The IgG/IgA Env ratio significantly correlated with increased risk of infection (decreased vaccine efficacy) [22]. In the presence of low vaccine-elicited IgA responses, either ADCC or NAb responses correlated with decreased risk of infection. ADCC responses were predominantly directed to the C1 conformational region of gp120. C1 on gp120 is a component of a target epitope for ADCC [23]. IgA antibodies elicited by RV144 could block C1 region-specific IgG-mediated ADCC (via natural killer cells) [22].

A sieve analysis identified two signatures of vaccine pressure within the V2 loop corresponding to sites 169 and 181. Intriguingly, vaccine efficacy (VE) against viruses matching the vaccine at position 169 was 48% whereas VE against viruses mismatching the vaccine at position 181 was 78%, supporting the hypothesis that vaccination-induced high V2 binding antibodies were associated with reduced risk of HIV-1 acquisition [24]. The explanation of a greater VE associated against mismatched HIV-1 with the sieve effect at site 181 remains unclear. It is speculated that vaccine-induced responses may have hindered HIV-1 infection with 181 variants, other explanations including involvement of other unidentified sequences near position 181 or inability of this variant to establish infection due to steric hindrance with vaccine-induced antibodies. The assessment of a T-cell based sieve effect in envelope V1/V2 revealed an association between an HLA class I allele and VE, suggesting that VE was restricted to A*02(+) participants and that IgA-C1 antibodies inhibited protective effects of other responses in A*02(−) participants [25].

RV305 explores systemic and mucosal immune responses elicited by late boosts (7–8 years post) administered to RV144 vaccine recipients. Another trial (RV306) recapitulating the RV144 regimen is expected to start in 2013 in Thailand and will explore the added value of boosts at 12 months, the immune responses in mucosal compartments and memory B cells. RV328 will evaluate AIDSVAX® B/E alone, recapitulating the Vax003 vaccination regimen to generate samples that will allow immunologic comparisons with RV144-like regimens containing ALVAC-HIV priming.

One of the main objectives for future vaccines is to counter HIV-1 variability. Antigenicity studies of the envelope used in RV144 suggest that certain epitopes were better exposed as a result of a non-HIV-1 sequence inserted into the HIV-1 envelope and likely elicited antibody epitope specificities in RV144 [26], in particular higher levels of V2 antibodies. Whether various envelope immunogens eliciting V2 antibodies are functional in a cross-clade manner and universal correlates of risk remains to be demonstrated. Vaccines utilizing a combination of consensus and transmitted-founder envelopes may be able to induce neutralizing responses with greater breadth and potency than single envelope immunogens [27]. In contrast, mosaic HIV antigens expressed by Ad26 vectors markedly augmented both the breadth and depth of antigen-specific CMI responses as compared with consensus or natural sequence HIV antigens in rhesus monkeys [28,29]. Heterologous prime-boost vectored vaccines (Ad26+MVA) encoding mosaic antigens are planned to soon enter clinical trial (RV307) in Thailand. The use of vectors such a ChAdV63 expressing conserved HIV-1 sequences [30] represents another promising approach now tested in humans [8], although not currently envisaged in Asia. Whether vaccine efficacy may prove to be strain-specific, region-specific or universal in populations with various modes of transmission remains to be demonstrated in future efficacy trials. Our current understanding of the immune correlates of protection suggests that no specific vaccine approach should be privileged over the other and that all reasonable vaccine approaches deserve to be pursued. 

In China, a plasmid DNA and a replication-competent Tiantan vaccinia HIV vaccine vector expressing HIV-1 CN54 CRF07_B’/C *gag-pol*, *env* and *nef* genes [31] is now in Phase II. Preliminary results suggest that both vaccines are safe and immunogenic [32]. An efficacy trial in MSM populations is now envisaged. 

### 2.2. Epidemiological Patterns in South-East and Eastern Asia

While in Sub-Saharan African the vast majority of HIV infections occur through heterosexual transmission [33], in SEA and Eastern Asia the epidemic patterns have evolved from heterosexual (female sex workers, new military recruits) now declining to predominantly anal transmission through unprotected intercourse in MSM and transgender (TG) populations [34,35,36,37]. Although previous studies suggested that in Thailand, Indonesia, and Myanmar, there was no significant decline in the prevalence of HIV epidemics in injecting drug users (IDU) [38], recent reports show that harm reduction programs have demonstrated a dramatic and beneficial impact on the epidemic in these populations [39].

The majority of HIV strains circulating in South East Asia with growing presence in China are represented by CRF01_AE with an increasing number of recombinant forms with B and C subtypes [40]. CRF01_AE dominates in Indonesia [41], Thailand (developed below), Cambodia [42], Laos, Myanmar, and Viet Nam [43,44]. In Malaysia, co-circulation of CRF01_AE and subtype B [45] has resulted in the emergence of CRF33_01B in approximately 20% of its HIV-1 infected individuals [46], now described in Indonesia [47]. We develop the situation of Thailand and China for their long-standing efforts in HIV vaccine development, and Myanmar, as a recently opened country after decades of isolation and with growing exchanges with China.

### 2.3. Thailand

Approximately 90% of incident infections in RV144 were CRF01_AE infections, a predominant circulating strain in Thailand and much of South East Asia. Among 390 volunteers who were deferred from enrolment in RV144 due to pre-existing HIV-1 infection using a multi-region hybridization assay, full genome sequencing and phylogenetic analyses showed the following subtype distribution: CRF01_AE: 91.7%, subtype B: 3.5%, B/CRF01_AE recombinants: 4.3%, and dual infections: 0.5%. CRF01_AE strains were 31% more diverse than those from the 1990s Thai epidemic that informed vaccine immunogen design. Sixty-nine percent of subtype B clustered with cosmopolitan Western B. Ninety-three percent of B/CRF01_AE recombinants were unique; recombination breakpoints analysis showed that these strains were highly embedded within the larger network that integrates recombinants from East/Southeast Asia. Forty-three to forty-eight percent of CRF01_AE sequences differed from the vaccine insert in Env V2 positions 169 and 181, which were implicated in vaccine sieve effects in RV144. Compared to the molecular picture at the early stages of vaccine development, the analysis of the molecular evolution of the HIV-1 Thai epidemic between the time of RV144 immunogen selection to the execution of the vaccine efficacy trial shows an overall increase in the genetic complexity of the Thai epidemic, increased distance to vaccine immunogens, and represent a clear example of viral evolution that occurred between immunogen design and vaccination for an efficacy trial [48]. Although the level of genetic complexity observed was consistent with the risk levels of a community-based cohort, the changes observed (expansion of the genetic diversity within CRF01_AE, increase in frequency and complexity of B/CRF01_AE recombinants, and shifts in the Western B/B’ balance) may have impacted the efficacy of the vaccine. The molecular epidemic changes that occurred between the time of vaccine design and efficacy trial should be carefully considered at the time of the analysis for future efficacy trials. It also suggests that the evolution of the molecular epidemic, although unavoidable, may be more limited in a region with relatively homogeneous and dominant HIV-1 strains, which may be more propitious for HIV vaccine efficacy testing.

In 2007, HIV prevalence among MSM in Bangkok and Chiang Mai was 30.7% and 16.9%, respectively, essentially unchanged from 2005 [35]. The HIV prevalence found in subsequent studies ranged from 5.5–28.3% with an incidence rate of 8.2 per 100 person-years [35,49], and 6% in Bangkok between 2006–2008 [36]. In a recent cohort study conducted in Pattaya, HIV incidence is 5.8 and 6.3 per 100 person-years among MSM and TG sex workers, respectively [50]. New MSM and TG cohort studies are now planned to prepare the conduct of future efficacy prevention trials.

### 2.4. Myanmar

Data on the HIV-1 epidemic in Myanmar are still scarce and of questionable representativeness. No HIV incidence data are available. According to the HIV sero-surveillance survey 2011 [51] and the progress report of the National Strategic Plan for HIV/AIDS 2011 [52], the number of female sex workers (FSW) were estimated between 45,000 and 62,000 (12,000–15,000 in Yangon and 7,800–11,000 in Mandalay) with an overall HIV prevalence of 9.4% (12% in direct FSW—defining themselves as sex workers and earn their living by selling sex and 6% in indirect FSW—for whom sex work is not the first source of income; Yangon 18%, high compared to the 2.5% in Bangkok; other cities 5–11%). HIV prevention programs now reach more than 76% of FSW and with more than 95% reported condom use at last sex. HIV-1 prevalence among FSW dropped drastically from >30% in 2006 to current figures. The number of MSM is estimated at 240,000, mostly in Yangon and Mandalay, with an overall HIV prevalence of 7.8% (Mandalay 9%, Yangon 5%), with more than 81% using a condom at last sex. As for FSW, we observe the same decreasing trend over the past five years. 

Importantly, the current HIV epidemic figures in FSW may offer a unique and perhaps, last opportunity to access high risk heterosexual populations for an HIV vaccine efficacy trial in SEA. However, this window of opportunity may narrow quickly as increased access to prevention services may improve [53]. Recent data on HIV subtypes circulating in Myanmar are limited to the Myanmar-China border, mostly in IDU populations [54,55,56]. However, the predominant circulating recombinant form in Myanmar remains CRF01_AE [40].

### 2.5. China

In the absence of interventions, HIV will spread very quickly in the MSM population [57] with an estimated reproductive ratio of 3.9 [58]. The proportion of MSM in the annually reported HIV cases increased from 12% in 2007 to 33% in 2009 [59]. A recent study showed an overall HIV prevalence of 4.9% with however considerable heterogeneity between provinces, with highest prevalence being up to 18% in southwestern provinces [60]. Approximately 25% of Chinese MSM are married [61,62] and 30% of these individuals have sex with a steady female partner, but with a low rate of condom use [63], constituting a dangerous bridge of HIV transmission from high-risk groups to the general population [64,65]. HIV incidence among MSM populations varies per province: 2.6 per 100 person-years in Beijing [66], 3.9% in Yunnan Province [67], 5.1% in Nanjing, Jiangsu Province [68], and 5.4% in Shenyang, Liaoning Province [69]. 

As early as 2006, CRF01_AE was found dominant (prevalence of 40.5%, 85.4% being acquired by sexual transmission) in Yunnan Province, in particular at the border with Myanmar [70]. Phylogenetic analysis indicates that the CRF01_AE sequences can be grouped into four clusters, suggesting that at least four genetically independent CRF01_AE descendants were circulating in China, of which two were closely related to the isolates from Thailand and Vietnam. Cluster 1 had the most extensive distribution in China. In North China, CRF01_AE clusters 1 and 4 are rapidly spreading in MSM [71]. In Yunnan, the distribution of HIV-1 strains in MSM was 71.4% CRF01_AE, and 28.6% CRF07_BC [67]. HIV-1 CRF01_AE accounted for 84% of the recent infections among MSM in Liaoning Province of northeastern China [72]. Recent studies give a different breakdown of recombinant forms: In Yunnan, CRF07_BC (18.9%), CRF08_BC (39.1%), CRF01_AE (22.4%), and URFs (subtype C, 5.9% and subtype B, 4.5%) [73]; *pol* sequences in newly diagnosed HIV-infected individuals from Dehong county, Yunnan, showed that subtype C accounted for 43.1%, unique recombinant forms for 18.4%, CRF01_AE for 17.7%, B for 10.7%, CRF08_BC for 8.4%, and CRF07_BC for 1.7% [74]; in Fujian, CRF01_AE (70.9%), C/CRF07_BC/CRF08_BC (5.8%), B/B’ (15.1%), and unique recombinant forms (8.1%) [75]. Similar trends are observed in Guizhou Province [76], Guangdong, Guangxi [77], Jiangxi and Hunan southern Provinces [78], Hong Kong [79] and Shanghai [80]. The HIV epidemic among MSM in China is expanding to Japan and illustrates the ongoing mixing of CRF01_AE and subtype B lineages unique to HIV-1 circulating in MSM populations in East Asia [81]. New CRF01_AE/B recombinants are now circulating among MSM [82,83]. These constantly evolving patterns illustrate the need to closely monitor the molecular HIV epidemic in potential target populations for HIV vaccine efficacy trials. Unless cross-protection can be demonstrated, the relevance of an HIV vaccine designed to targeting HIV-1 B’/C recombinants in populations mostly infected with CRF01_AE may be questionable, while a CRF01_AE-based vaccine such as the one tested in Thailand would seem more appropriate.

### 2.6. Impact of a HIV Vaccine on the HIV Epidemic and Cost-Savings

An HIV vaccine with VE 50% and with 30% coverage of low-and-middle income country populations could avert between 5.2 and 10.7 million new HIV infections between 2020 and 2030. Based on the WHO 2010 Guidelines for Antiretroviral Therapy and the 2010 antiretroviral drug (ARV) costs for low- and middle-income countries ($155 for first-line and $1,678 for second-line and assuming a second-line ARV decline to $980 by 2015, plus costs of diagnostics and monitoring tests of $180 and service delivery of $176 per patient, per year), an HIV vaccine would save between $46 billion to $95 billion in averted costs of ART provision alone, depending on the characteristics of the vaccine and population coverage levels achieved [84]. In a scenario in which HIV/AIDS programming is scaled up to the UNAIDS Investment Framework targets [85], the number of new HIV infections and costs averted would be between 1.6 and 3.3 million and $14 billion and $29 billion, respectively [86]. A modeling analysis for the province of Sichuan, China, described similar cost-saving patterns [87].

A vaccine with rapidly waning protection could have a substantial impact on the epidemic in Thailand [88,89]. Factors influencing the impact of such a vaccine include risk compensation, vaccine efficacy, and duration of protection. Due to the short duration of effect with the RV144 regimen, for example, large numbers of vaccinations would be needed to maintain high population coverage levels.

Over the past 15 years, Thailand and China have both considerably invested in HIV vaccine clinical development and (for China) pilot manufacturing [12], and are now spearheading a regional effort that may one day lead an HIV vaccine to licensure. Regional markets are defined epidemiologically and economically in a booming region with genuine regional manufacturing capacity and advanced knowledge-based industry. Vaccine manufacturers may see investment in HIV vaccines as a way to secure access to private vaccine markets for other licensed or developmental products [90]. Infrastructure, know-how, management and leadership and international and regional, in particular through public-private partnerships (PPP) such as the pox-protein PPP (P5) [91] and the AIDS Vaccine for Asia Network (AVAN) [92,93,94,95], collaboration are key elements of success.

## 3. Challenges

Because the modest efficacy conferred by the RV144 regimen was observed in a mostly heterosexual Thai population at low risk for HIV infection and with low HIV incidence, follow-up efficacy studies to verify this result in a similar population would necessitate prohibitively large numbers of volunteers. Efficacy trials would be smaller and less costly if implemented in higher risk populations with high HIV incidence. It is argued that vaccine protection might be easier to achieve in high-risk populations with predominantly heterosexual transmission such as those found in Africa [33,96], in contrast to MSM populations with rectal transmission, with the highest risk mode of sexual HIV transmission at 1:20–1:300 infections per exposure [97]. So far, high-risk heterosexual populations suitable for efficacy trials have not been identified in SEA. The rampant epidemic in Sub-Saharan Africa accounting for 69% of people living with HIV worldwide [98] has understandably resulted in a shift in priority for HIV vaccine investments to Sub-Saharan Africa, in particular southern Africa, with less emphasis on Asia. 

Identification of intermediate- to high-risk populations with predominant heterosexual transmission in Asia deserves greater consideration for future efficacy trials [8,12] as a means of improving trial feasibility and generalizability. For example, the HIV prevalence reported among the female sex workers in Yangon is the highest (18%) in SEA [51]. This opportunity to identify such populations may however rapidly wane with the scale-up of HIV prevention strategies in this country [33]. 

The success of harm reduction programs has yielded decreasing HIV incidence in IDU [38,39]. For example, only 7 of 1,157 HIV-seronegative IDU acquired HIV over a two-year follow-up in HPTN 058 trial conducted in Xinjiang and Guangxi Provinces, China, and Chiang Mai, Thailand [12,99,100]. Consequently, HIV vaccine efficacy trials in IDU are unlikely to proceed. Moreover, a high proportion of new circulating recombinant forms are now found in IDU, as illustrated by CRF01_AE/B’/C recombinants (42.6%) in northern Myanmar at the border with China [54,55,56] and India [101]. Taken together with the high burden of multiple transmitted/founder variants in IDU [102], it would be unlikely that vaccines eliciting humoral and and/or cell-mediated T-cell responses of limited breadth would be efficacious in IDU populations. This pattern may also become true in other high-risk groups due to increased mixing of populations that may result in increased proportions of HIV-1 circulating recombinant forms, in particular at the borders with China and India.

HIV prevention research has shifted to the evaluation of combination prevention programs whereby biomedical, behavioral, and structural interventions are implemented concurrently to maximize synergies among interventions [103]. New prevention strategies to control the epidemic and prevent new infections, including pre-exposure prophylaxis [104], antiviral treatment as prevention [105,106], and topical microbicides [107], are now being actively developed. PrEP reduced the risk of HIV infection by an average of 44% (73% among high adherers) in HIV negative MSM and TG women who participated in the iPrEx, a clinical trial testing a daily oral dose of the antiretroviral emtricitabine/tenofovir drug combination [108]. Following these encouraging results, a recent study suggested that despite multiple challenges, MSM in Thailand would be willing to take PrEP, even if they had to experience inconvenience and expense [109]. Among MSM living in Beijing, despite low awareness of PrEP, 68% were willing to accept PrEP [110]. However, such hypothetical projections may not be matched by the daily reality, as suggested by the cascade of PrEP volunteers in the OLE extension of iPrEx trial [111]. The rationale and theoretical aspects of an efficacy trial of combination prevention modalities such as HIV vaccine and PrEP acting in synergy have been described [112,113]. The conduct of such trials remains however hypothetical and would require high adherence to PrEP, larger sample sizes, be more costly, and complicate the regulatory approval process to licensure.

The licensure of HIV vaccines raises several issues that are yet to be addressed. For example, would an HIV vaccine efficacious in MSM be licensed for heterosexual populations if there were no longer the opportunity to test this vaccine in such Asian populations? What would be the requisites of SEA countries for licensure of an efficacious HIV vaccine tested in Thailand only? What would be the requirements for licensure of a vaccine manufactured in Thailand but whose licensure trial lots have been manufactured outside Thailand? What would be the requirements for licensing this vaccine for adolescents? Safety and immunogenicity bridging studies would be required. Efficacy trials in adolescents would provide much needed data in advance of implementing vaccination in this population, however, social and regulatory acceptability represent serious challenges [114]. Public Health Policy, pharmacovigilance, and epidemiological surveillance systems should be put in place before the marketing of the vaccine. A close collaboration from the inception of licensure trial designs between scientists, Public Health Authorities and National Regulatory Agencies is therefore highly desirable and recommended. The use of vaccines expressing multiple HIV proteins, in particular, envelope protein subunits, may increase the risk of vaccine-induced seropositivity (VISP) in some proportion of vaccinated individuals when using routine HIV diagnosis serological tests [115]. This has raised concerns at individual and public health levels [116] as it may seriously complicate the epidemic surveillance of a country if easy-to-perform and cheap tests are not made available [117,118]. 

Vaccine development has become frustratingly slow. Several factors contribute to this situation including lengthy convoluted approval processes, complicated multi-product vaccine regimens, and timely availability of clinical lots of candidate vaccines, in particular, envelope subunit proteins formulated with potent adjuvants. This situation needs careful consideration and urgent remedies as it may cause communities, donors and scientists experiencing time or budget constraints to waiver in their commitment to HIV-1 vaccine development. The uncertain commitment of pharmaceutical companies and donors for SEA HIV vaccines has led to consideration of sub-optimal strategies including the use of soluble envelope subunit proteins derived from other HIV-1 subtypes (for example, subtype C protein to be used in Africa) that may or may not be suitable for protection against HIV acquisition in SEA, as cross-clade functional reactivity and protection remain unproven. The manufacturing of an envelope subunit protein in SEA, a scenario now being actively pursued, may result in accelerating the availability of this vaccine component for efficacy testing while building vaccine manufacturing capacity for non-HIV vaccines. This will require political and long-term funding commitments. 

Availability does not guarantee but may impact uptake. Socio-cultural and structural contexts of HIV vaccine acceptability among most-at-risk populations were studied in Thailand. Crosscutting challenges for HIV vaccine uptake such as social stigma, discrimination in healthcare settings and out-of-pocket vaccine costs emerged in addition to population-specific barriers and opportunities [119]. HIV vaccine acceptability and risk compensation among high-risk MSM and TG ranged from 31.6–73.8 on a 100-point scale (mean = 58.3). VISP had the greatest impact on acceptability, followed by efficacy, vaccine-related side effects, duration of protection, out-of-pocket costs and social saturation. Over one-third (34.6%) reported intention to increase post-vaccination risk behaviors in response to a highly efficacious HIV vaccine [120].

## 4. Conclusions

While challenges are real, epidemiologic patterns and the dynamic of clinical development in Thailand and China represent tremendous opportunities to bring an HIV-1 vaccine to licensure. Better understanding of the immune correlates of protection remains a key element to direct the vaccine effort to counter heterogeneous circulating virus strains among populations with different modes of HIV transmission. The molecular epidemic changes between the time of vaccine design and efficacy trial should be monitored and carefully considered in analysis for future efficacy trials. If emphasis has been recently given to the possible involvement of V2 antibodies in protection against HIV acquisition, other approaches such as immunogens capable of inducing broadly neutralizing antibodies along with cell-mediated immune responses of greater breadth and depth (mosaic and conserved sequences) should be aggressively pursued. While conducting efficacy trials in MSM and TG seems the likely scenario for Asia, genuine efforts to identifying heterosexual populations suitable for such trials, though difficult, should be pursued. Engaging communities, regulatory and public health authorities in a mutually constructive and educational dialogue seems essential to address and help overcome these challenges as well as to prepare for success and access, or failure. A vaccine countering CRF01_AE is a priority for the region. An Asian vaccine against expanding B/E or BCE recombinant forms should be actively pursued. These new avenues will only be made possible through the conduct of large-scale efficacy trials, interdisciplinary teams, international collaborations, and strong political and community commitments.
